# Ultrafast Light‐Driven Electronic and Structural Changes in LaFeO_3_ Perovskites Probed by Femtosecond X‐Ray Absorption Spectroscopy

**DOI:** 10.1002/adma.202502932

**Published:** 2025-05-15

**Authors:** Masoud Lazemi, Fabian J. Mohammad, Sang Han Park, Abhishek Katoch, Hans J.F.A. Blankesteijn, Andrés R. Botello‐Méndez, Emma van der Minne, Yorick A. Birkhölzer, Iris C. G. van den Bosch, Ellen M. Kiens, Christoph Baeumer, Gertjan Koster, Soonnam Kwon, Uwe Bergmann, Frank M. F. de Groot

**Affiliations:** ^1^ Materials Chemistry and Catalysis Debye Institute for Nanomaterials Science Utrecht University Universiteitsweg 99 Utrecht 3584 CG The Netherlands; ^2^ MESA+ Institute for Nanotechnology University of Twente P.O. Box 217 Enschede 7500 AE The Netherlands; ^3^ Pohang Accelerator Laboratory Pohang Gyeongbuk 37673 South Korea; ^4^ Department of Chemistry Yonsei University Seoul 03722 South Korea; ^5^ Department of Physics University of Wisconsin–Madison Madison WI 53706 USA

**Keywords:** density funcational theory, femtosecond X‐ray absorption spectroscopy, LaFeO_3_, multiplet calculations, X‐ray free‐electron laser

## Abstract

Conducting real‐time, element‐specific studies of photo‐excited systems is a long‐standing challenge. The development of X‐ray free‐electron lasers (XFELs) has paved the way for the emergence of a promising technique: femtosecond X‐ray absorption spectroscopy (fs‐XAS). This powerful technique reveals electronic and geometric characteristics, providing unprecedented insight into their dynamic interactions under nonequilibrium conditions. Herein, the fs‐XAS technique is employed at PAL‐XFEL to unravel light‐driven ultrafast electronic and structural changes in epitaxial lanthanum iron oxide (LaFeO_3_) thin films. Density functional theory (DFT) and multiplet calculations are utilized to expound on the experimental results. The analyses reveal that photoexcitation initially induces high‐ and intermediate‐spin Fe^2+^ states through ligand‐to‐metal charge transfer (LMCT), followed by polaron formation. It is demonstrated that the reduced overlap between the oxygen 2*p* and iron 3*d* orbitals accounts for all experimental observations, including 1) the XAS shifts to lower energies, 2) the decrease in the crystal field splitting, and 3) the relatively larger shifts observed in the oxygen 1*s* XAS.

## Graphical abstract

Femtosecond X‐ray absorption spectroscopy (fs‐XAS) at PAL‐XFEL captures ultrafast electronic and structural dynamics in epitaxial LaFeO_3_ thin films. Combined with DFT and multiplet calculations, the study reveals photoinduced ligand‐to‐metal charge transfer, followed by polaron formation and reduced oxygen 2*p*‐iron 3*d* hybridization, explaining key spectral changes.

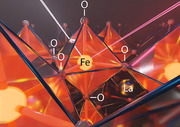

## 1 Introduction

Developing advanced photoanode materials with tailored properties is crucial for enhancing photoelectrochemical water splitting. Titanium dioxide (TiO_2_) and hematite (α‐Fe_2_O_3_), thus far, have been the most studied metal oxides as photoelectrodes.^[^ ^1^ ^]^ However, their large bandgaps and extremely short charge carrier diffusion lengths pose significant challenges to achieving higher solar‐to‐hydrogen conversion efficiencies. Lanthanum transition‐metal oxides (LaTMO_3_) have garnered considerable attention as rising candidates.^[^ ^2^ ^]^ Varying the TM enables the creation of a myriad of lanthanum‐based perovskites with distinct chemical and physical properties as well as photoelectrochemical performance. Understanding and optimizing the performance of such materials necessitate advanced spectroscopy techniques capable of probing their ultrafast electronic and structural dynamics.

The emergence of X‐ray free‐electron lasers (XFELs) has made X‐ray absorption spectroscopy (XAS) accessible for time‐dependent studies on the femtosecond (fs) time scale (fs‐XAS).^[^ ^3^, ^4^, ^5^, ^6^, ^7^, ^8^, ^9^ ^]^ A wide range of intriguing applications beyond static synchrotron experiments are heralding the dawn of a new era in many scientific disciplines.^[^ ^10^ ^]^ Our group has recently reported the hole transport dynamics in TiO_2_ in a direct and real‐time manner.^[^ ^4^ ^]^ Besides TiO_2_, we have reported various fs‐XAS studies on photoelectrocatalytic materials, including MoTe_2_, α‐Fe_2_O_3_, CeO_2,_ and CuWO_4_.^[^ ^3^, ^11^, ^12^, ^13^, ^14^ ^]^

Despite extensive studies on the photocatalytic activity and ground‐state properties of lanthanum iron oxide (LaFeO_3_), prior investigations have primarily relied on static spectroscopic or electrochemical techniques, lacking direct insight into carrier dynamics and excited‐state processes.^[^ ^15^, ^16^, ^17^, ^18^, ^19^ ^]^ Such approaches are unable to capture the ultrafast processes and transient electronic structures that underlie key efficiency‐limiting mechanisms, including carrier recombination and polaron formation. In particular, the lack of element‐ and orbital‐specific time‐resolved measurements has hindered a detailed understanding of charge localization and spin state evolution following photoexcitation.

Herein, we focus on the fs‐XAS study of photoexcited LaFeO_3_ epitaxial thin films at the PAL‐XFEL.^[^ ^20^ ^]^ We and other groups have reported the promising activity of LaFeO_3_ for the oxygen evolution reaction as a photoanode.^[^ ^15^, ^18^ ^]^ However, to the authors’ knowledge, the dynamics of photo‐excited carriers in LaFeO_3_ have not yet been explored. After discussing the ground state (GS), excited state (ES), and transient (TR) XAS spectra, as well as kinetic traces at the oxygen 1*s* (K) edge of LaFeO_3_, we employ density functional theory (DFT) calculations to interpret the spectra. We then analyze similar spectra at the iron 2*p*_3/2_ (L_3_) edge and corroborate them with multiplet calculations. We demonstrate how photoexcitation initially induces high‐ and intermediate‐spin Fe^2+^ states through ligand‐to‐metal charge transfer (LMCT) following polaron formation. Our quantitative analysis of the observed fs‐XAS spectra provides detailed insights into the mechanisms underlying the dynamics that follow the photoexcitation of this promising photoelectrocatalyst.

## 2 Results and Discussion

**Figure 1:**
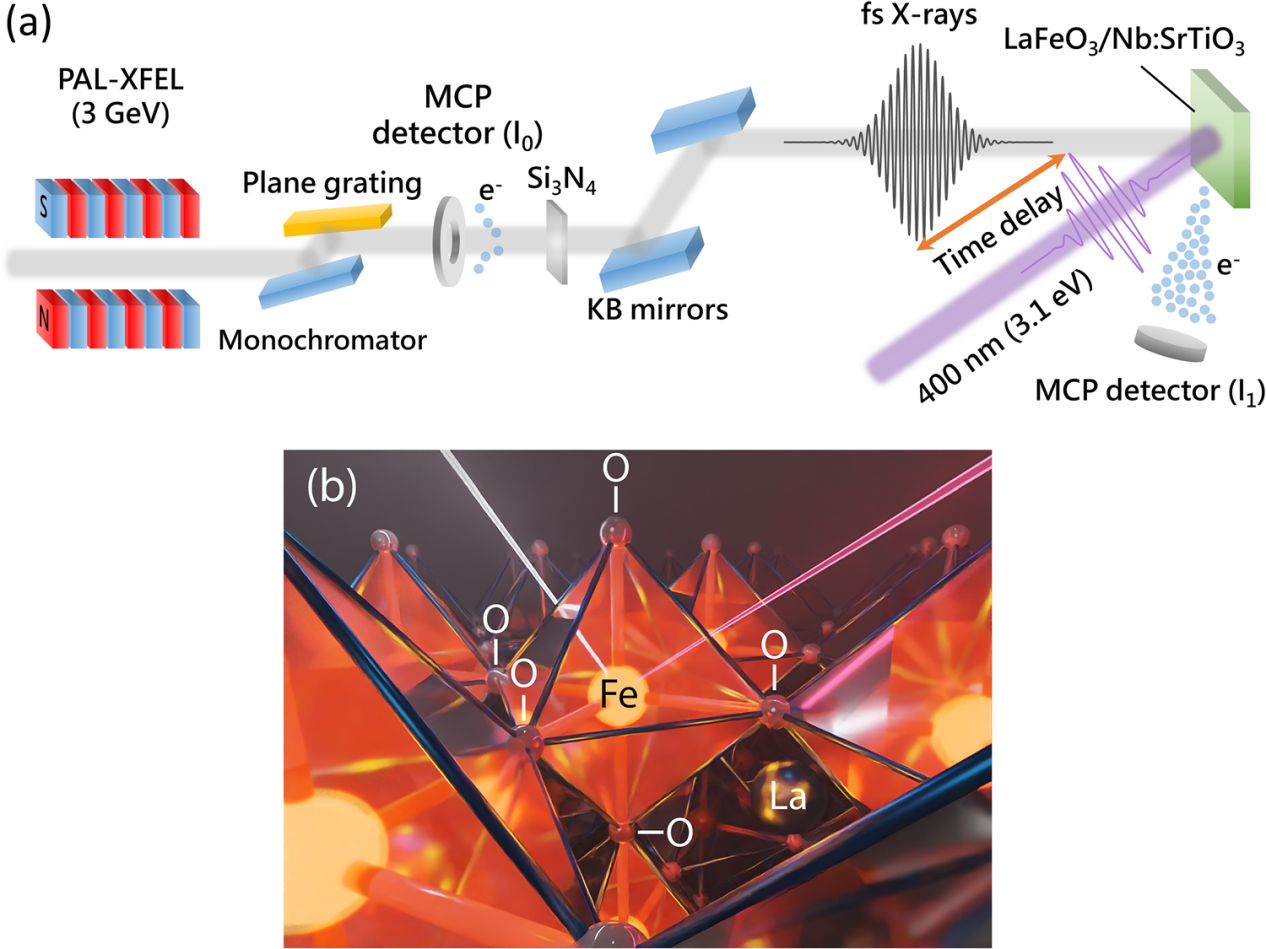
a) The pump‐probe XAS experimental setup at the Soft X‐ray Scattering and Spectroscopy (SSS) beamline of PAL‐XFEL b) Schematic of the LaFeO_3_ perovskite structure.

**Figure** **1**a,b depict the experimental setup used in our pump‐probe fs‐XAS experiment to investigate the photoexcited state of LaFeO_3_, which has an optical band gap of ≈2.0 eV.^[^ ^21^ ^]^ The sample was excited by a 400 nm (3.1 eV) optical pump laser with an 80‐fs pulse width, followed by probing at the oxygen 1*s* (K) and iron 2*p*_3/2_ (L_3_) edges, using 80‐fs X‐ray pulses.

### 2.1 Ground‐State and Transient Oxygen 1s XAS Spectra

#### 2.1.1 DFT Calculations

**Figure 2:**
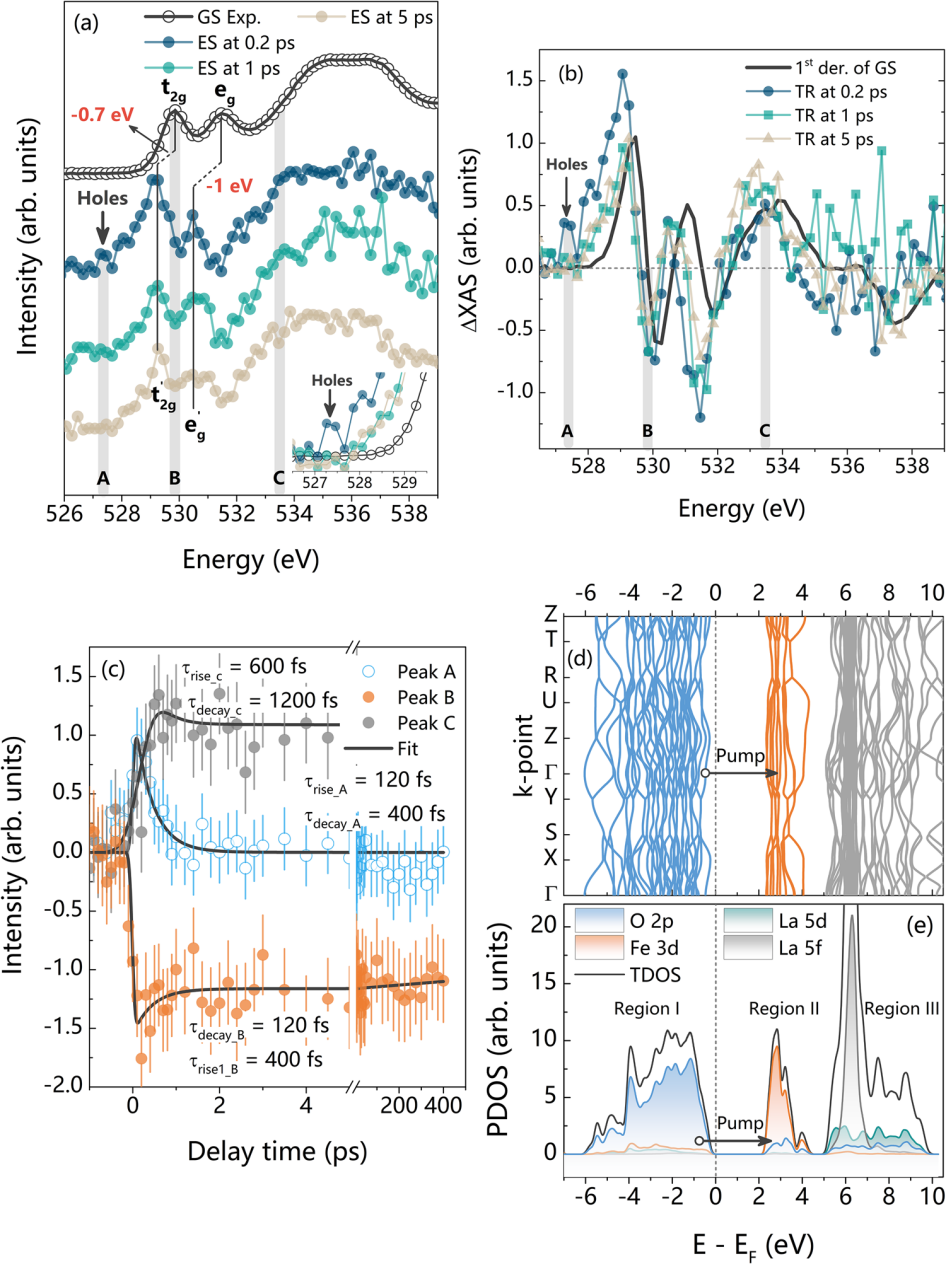
Comparative analysis of oxygen 1*s* fs‐XAS of LaFeO_3_ a) ground state (GS) and excited state (ES) spectra at 0.2, 1, and 5 ps. The energy shift for the *t_2g_* and *e_g_* peaks are −0.7 and −1 eV, respectively. The ES spectra were derived by Equation S5 (Supporting Information), $ES\;\left(\lambda ,t\right)=\frac{1}{\alpha }\;TR\left(\lambda ,t\right)+GS\left(\lambda ,0\right)>0$, considering α  =  0.1. The inset shows the enlarged region between 526.5 and 529.5 eV. The transient peak, marked by an arrow, is attributed to the ultrafast transition of oxygen 1*s* electrons to oxygen 2*p* hole states. Similar spectra with different α values are plotted in Figure S3 (Supporting Information). b) The 1st derivative of GS and transient (TR) spectra at 0.2, 1, and 5 ps. For clarity, the TR spectra were multiplied by 5, while the 1st derivative of GS was divided by 3. c) Kinetic traces of transient XAS at Peak A (526.86 eV), Peak B (529.86 eV), and Peak C (533.46 eV), fitted using Equation S6 (Supporting Information). The error bars are depicted as vertical lines. d) Calculated electronic band structure of LaFeO_3_ along high‐symmetry points e) Calculated projected density of states (PDOS) of LaFeO_3_ as a function of E – E_F_, depicting the total density of states (TDOS), lanthanum 5*d* and 5*f*, iron 3*d*, and oxygen 2*p* states.

The ground‐state (GS) oxygen 1*s* XAS spectrum of LaFeO_3_ is depicted in **Figure** **2**a. The shape of the oxygen 1*s* XAS can be approximated by the unoccupied states with the oxygen *p* symmetry in the presence of a core hole.^[^ ^22^, ^23^ ^]^ We have performed DFT calculations to interpret the XAS spectra, as shown in Figure 2d,e. LaFeO_3_ has a 3*d* ^5^ high‐spin 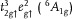 configuration, suggesting that the unoccupied iron 3*d* spectral density will mainly have *t*_2*g*↓_ and *e*_*g*↓_ characters.^[^ ^24^, ^25^, ^26^ ^]^ Hence, in Figure 2a, the features at 529.86 eV (hereafter Peak B) and 531.45 eV are attributed to transitions into iron *t*_2*g*↓_ and *e*_*g*↓_ bands, respectively.

The *t_2g_* and *e_g_* peaks are assigned to hybridization between oxygen 2*p* and iron 3*d* states. It should be noted that there is a subtle difference between the *t_2g_* and *e_g_* splitting compared with crystal field splitting (CFS). The energy difference between the *t_2g_* and *e_g_* at the oxygen 1*s* XAS was found to be 1.6 eV (Figure S1 and Table S1, Supporting Information), which agrees well with previous reports.^[^ ^27^, ^28^, ^29^ ^]^ Nonetheless, this value is smaller than CFS resulting from iron 2*p* XAS calculations, i.e., 1.8 eV, as reported in Section 2 and elsewhere.^[^ ^24^ ^]^ The underlying reason for these differences is that the oxygen 1*s* core hole reduces the splitting between the two peaks. In iron oxide compounds, the core hole potential pulls down the *t_2g_* and *e_g_* states to the bottom of their bands. The *e_g_* band is broader than the *t_2g_* band, and the energy difference between the centers of the *t_2g_* states and *e_g_* states is reduced to a value of 1.6 eV in the GS oxygen 1*s* XAS. The same effect is visible in Fe_2_O_3_, where the average *t_2g_*–*e_g_* splitting is reduced to a 1.2 eV splitting in the oxygen 1*s* edge.^[^ ^23^, ^30^, ^31^ ^]^

In Figure 2d,e, we identify three distinct regions by setting the Fermi level at the top of the valence band (VB):

- Region I: dominant contributions from the oxygen 2*p* orbitals (between −6 and 0 eV)
- Region II: antibonding iron 3*d* and oxygen 2*p* states (between 2 and 4.5 eV)
- Region III: antibonding lanthanum 5*d* and oxygen 2*p* states (between 5 and 10 eV)

The band structure between 2.0 and 2.5 eV is almost nondispersive across momentum space due to the localized nature of the unoccupied Fe^3+^ minority spin *t_2g_* states. The states between 2.5 and 4.5 eV are primarily derived from the minority spin iron 3*d* states, and their overlap with the oxygen 2*p* states results in a larger bandwidth and dispersion than for the *t_2g_* states.

### 2.2 Excited State, Transient Spectra, and Kinetic Traces

#### 2.2.1 Region I: Oxygen 2*p* States

We acquired the kinetic traces below the oxygen 1*s* threshold energy at Peak A (526.86 eV) to examine the behavior of holes upon photoexcitation, as depicted in Figure 2a,c. According to our DFT calculations in Figure 2d,e, Peak A resides in Region I, with dominant contributions from the oxygen 2*p* orbitals. This assignment is consistent with earlier DFT‐based theoretical studies on similar transition‐metal oxides, which attribute pre‐edge features in oxygen K‐edge XAS to oxygen 2*p* states.^[^ ^21^, ^29^ ^]^

In this energy range, the oxygen 2*p* orbitals are not only involved in metal–oxygen bonding but also play a significant role in forming ligand‐hole states upon photoexcitation. These hole states are signatures of LMCT, where an electron from oxygen 2*p* is transferred to an iron 3*d* state. This is further supported by the transient spectrum (see inset of Figure 2a), which shows a distinct increase in intensity at Peak A shortly after excitation. This transient feature indicates an increase in the unoccupied oxygen 2*p* character near the valence band edge, consistent with the formation of photoinduced holes.

This interpretation is in line with our previous work on α‐Fe_2_O_3_, where we assigned a similar pre‐edge feature at 527.6 eV to transitions involving oxygen 2*p* states.^[^ ^12^ ^]^ In that system, we observed a decay constant of τ_decay_ = 200 fs, which is attributed to rapid hole recombination. In contrast, the kinetic trace in Figure 2c for LaFeO_3_ reveals a slower decay time of τ_decay_A_ = 400 fs, indicating longer‐lived hole states and suggesting more favorable charge separation in LaFeO_3_. The longer hole lifetime in LaFeO_3_ implies a more significant opportunity for the photogenerated holes to participate in oxidation reactions, thereby enhancing its potential as a photoanode material for the oxygen evolution reaction (OER). The kinetic constant for the rising (decaying) region of Peak A (Peak B) around time zero provides the instrumental resolution, which is 120 fs (Figure 2c). This aligns well with the resolution that we previously achieved for TiO_2_, Fe_2_O_3_, CuWO_4_, and CeO_2_ at the same beamline.^[^ ^4^, ^11^, ^12^, ^13^, ^32^ ^]^

#### 2.2.2 Region II: Antibonding Fe 3*d* and Oxygen 2*p* States

The oxygen 1*s* XAS in Region II manifests the unoccupied iron 3*d* orbitals. In other words, XAS is proportional to the oxygen character of the unoccupied iron 3*d* orbitals.^[^ ^22^, ^23^ ^]^ In LaFeO_3_, each iron atom is octahedrally coordinated by six oxygen atoms (Figure 1b), resulting in the crystal field splitting of iron 3*d* orbitals into *t_2g_* and *e_g_* states, as expected in an octahedral ligand field. As discussed in Section 1.1, Peak B and the neighboring features in Region II are attributed to these anti‐bonding *t_2g_* and *e_g_* orbitals. Specifically, Peak B corresponds predominantly to hybridized iron 3*d* *t_2g_*–oxygen 2*p* states, while the shoulder at higher energy reflects transitions into *e_g_*‐derived states. These assignments are supported by our calculated projected density of states (PDOS) results (Figure 2e), where the iron 3*d* density splits into two sub‐bands consistent with the *t_2g_* and *e_g_* separation. Previous studies employing oxygen K‐edge XAS have reported comparable orbital assignments in perovskite oxides.^[^ ^22^, ^23^ ^]^

The comparison of ground state (GS) and excited state (ES) spectra in Figure 2a,b reveals two key spectral changes in Region II following photoexcitation:

- A redshift of both *t_2g_* and *e_g_* peaks (−0.7 and −1.0 eV, respectively), indicative of changes in the local crystal field and bonding environment.
- A reduction in XAS intensity at these peaks can be attributed to the population of unoccupied iron 3*d* bands by photoexcited electrons. The redshift of the *t_2g_* and *e_g_* peaks indicates that the corresponding antibonding states in the excited state (ES) are less anti‐bonding than in the ground state (GS). This suggests an increased contribution from iron 3*d* orbitals and a reduced contribution from oxygen 2*p* orbitals in these bands. As the oxygen 2*p* character decreases, the transition probability at the oxygen K‐edge diminishes, resulting in a lower XAS intensity.

#### 2.2.3 Region III: Antibonding La 5*d* and Oxygen 2*p* States

Region III encompasses Peak C (533.46 eV), which is assigned to lanthanum 5*d*–oxygen 2*p* antibonding states (Figure 2d,e). The observed rise in Peak C following photoexcitation in Figure 2c, with a kinetic time constant of ≈600 fs, suggests that photoexcitation induces a delayed modification of the lanthanum–oxygen bonding environment. This occurs relative to the faster hole dynamics in the oxygen *2p* and iron 3*d* orbitals (Region I). This delay, similar to the 400‐fs decay time at Peak A, reflects structural relaxation or polaron formation.

Importantly, no significant transient changes are observed in the lanthanum 3*d* fs‐XAS spectra (Figure S4, Supporting Information), indicating that the lanthanum 4*f* and core states remain unaffected by photoexcitation. This is consistent with the known weak hybridization between La 4*f* states.^[^ ^29^ ^]^ The La atoms act primarily as “spectators,” and the observed dynamics in Region III are better understood as an indirect consequence of changes in the iron–oxygen sublattice rather than direct excitation of lanthanum orbitals.

### 2.3 Iron 2*p*_3/2_ fs‐XAS Spectra

**Figure 3:**
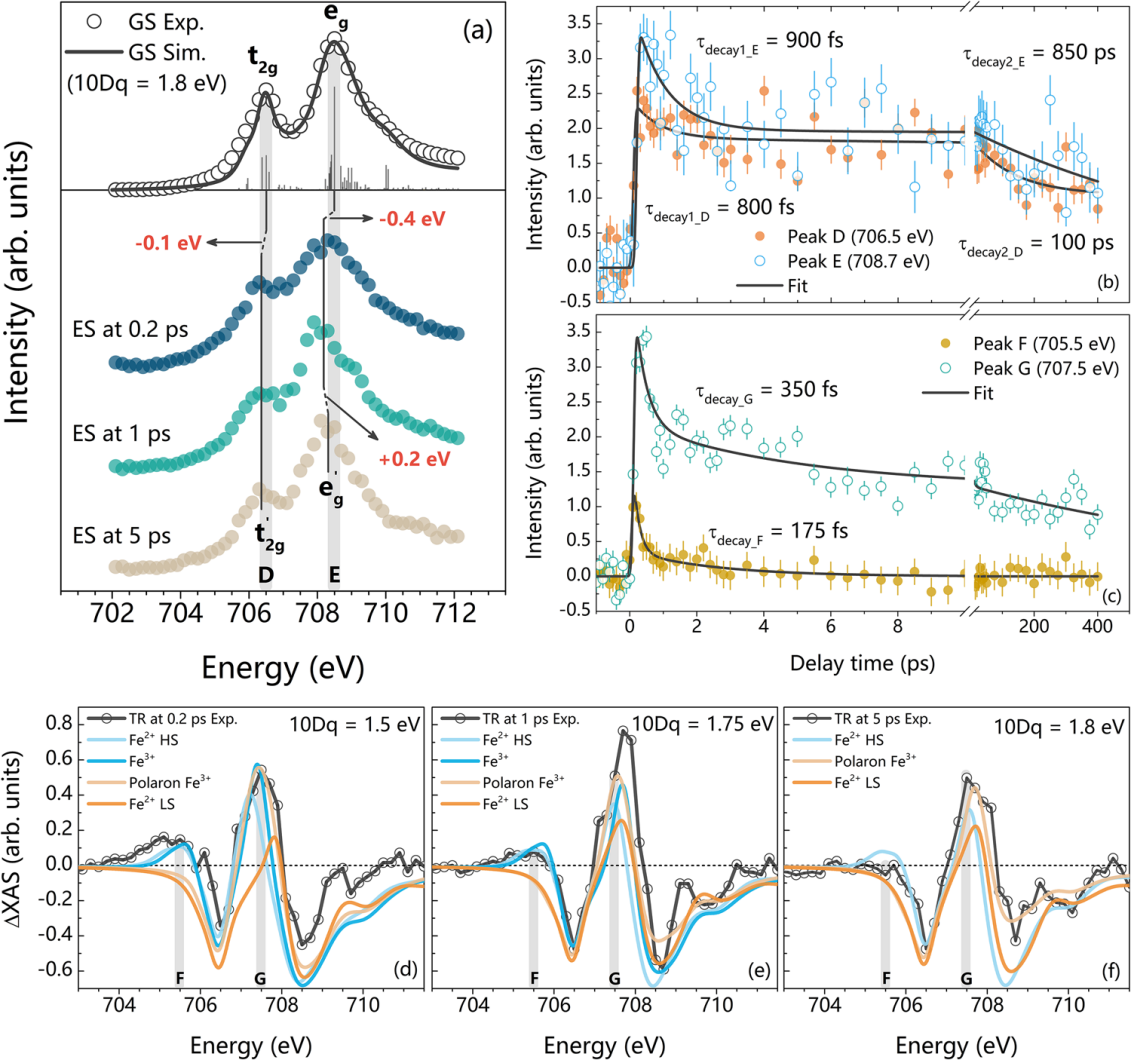
Comparative analysis of iron 2*p*_3/2_ fs‐XAS of LaFeO_3_ a) ground state (GS) and excited state (ES) spectra at the delay times of 0.2, 1, and 5 ps. The sticks represent crystal field multiplets calculated by CTM4XAS. The GS spectrum was calculated using 10Dq = 1.8 eV. The *t_2g_* and *e_g_* peaks shift −0.1 and −0.4 eV, respectively. The ES spectra were derived using Equation S5 (Supporting Information), considering α  =  0.1. The similar ES spectra with different α values are plotted in Figure S7 (Supporting Information). b) Kinetic traces of transient XAS at Peak D (706.5 eV) and Peak E (708.7 eV). c) Kinetic traces of transient XAS at Peak F (705.5 eV) and Peak G (707.5 eV). The error bars are depicted as vertical lines. d–f) Experimental and simulated transient (TR) spectra at the delay times of 0.2, 1, and 5 ps, considering Fe^2+^ high‐spin (HS), Fe^3+^, polaron Fe^3+^, and Fe^2+^ low‐spin (LS).

In the 2*p* XAS process, each 3*d* ^N^ state is excited to a final 2*p* ^5^3*d* ^N+1^ state.^[^ ^22^ ^]^ Our previous works include several comprehensive reviews of 2*p* XAS of 3*d* transition metal systems in refs. [30, 33, 34, 35]. These transitions are strongly influenced by both core‐hole effects and multiplet interactions arising from the higher‐order electron‐electron interactions between the 2*p* core hole and the 3*d* valence electrons. In general, in the iron 2*p* XAS spectrum of LaFeO_3_, the separation between the two peaks results from the final‐state spin‐orbit splitting of the core 2*p* states to 2*p*_3/2_ and 2*p*_1/2_ peaks from where the electrons are excited.^[^ ^22^ ^]^ Note that, in the initial state, these states are degenerate. As depicted in **Figure** **3**a, we focus on the 2*p*_3/2_ region, which itself exhibits further splitting due to the crystal field interaction—the splitting of Fe 3*d* orbitals into *t_2g_* and *e_g_* levels under the octahedral coordination of oxygen atoms. This crystal field splitting (CFS) manifests as distinct spectral features in the iron 2*p*_3/2_ XAS and serves as a sensitive probe of the local electronic structure and oxidation state of iron.

The iron 2*p* XAS spectra can be simulated by calculating the crystal field multiplets in octahedral symmetry.^[^ ^22^, ^36^ ^]^ We have developed a theoretical approach based on multiplet calculations to comprehend the time evolution of the iron 2*p*_3/2_ XAS spectra. This approach explicitly incorporates spin‐orbit coupling, crystal field effects, and LMCT. Figure 3d–f illustrates the calculated transient (TR) spectra for different delay times: 0.2, 1, and 5 ps. The GS is 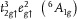 with all the same spin direction. Therefore, the 6^th^ excited 3*d* electron is spin‐down. The final state is thus $t_{2g↑}^{3}e_{g↑}^{2}$ + $t_{2g↓}^{1}$ + L, where L denotes a ligand‐hole. The presence of the ligand hole reflects the hybridized nature of the excited state, resulting in new electronic transitions and spectral redistribution. Moreover, photoexcitation leads to a parity inversion. Since the 3*d* ^5^ ground state has odd parity, the optically excited 3*d* ^6^ L configuration is of even parity and thus differs from the odd‐parity 3*d* ^6^ L states that contribute to the ground state.

We will now discuss the observations at the iron 2*p*_3/2_ edge reported in Figure 3.

#### 2.3.1 Ground State (GS)

The GS spectrum in Figure 3a features notably sharp multiplets and can be theoretically simulated by assuming a high‐spin $t_{2g↑}^{3}e_{g↑}^{2}$ (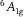) ground state with 10Dq = 1.8 eV.^[^ ^24^ ^]^ This indicates a stable, well‐defined structure where the iron–oxygen bond lengths and the surrounding lattice are in equilibrium. A comparison between the experimental and calculated GS spectra (Figure S5, Supporting Information) validates this assignment, showing that the GS of LaFeO_3_ is mainly 3*d* ^5^ ($t_{2g↑}^{3}e_{g↑}^{2})$ high‐spin, consistent with our previous findings.^[^ ^11^ ^]^

#### 2.3.2 Immediate Response (0.2 ps)

At 0.2 ps after photoexcitation, the system enters a highly non‐equilibrium state, where rapid electronic redistribution alters the local electronic environment. The reduction in crystal field splitting (CFS) from 1.8 to 1.5 eV (Figure 3d) is indicative of a weakened ligand field strength due to transient changes in iron–oxygen orbital overlap. This is a direct consequence of the excitation of electrons from oxygen 2*p*‐derived valence band states to iron 3*d* conduction band states, resulting in LMCT. The presence of LMCT‐driven high‐spin and intermediate‐spin Fe^2+^ states is supported by the observed transient spectral features at 0.2 ps, with a characteristic decay constant of 350 fs (Figure 3c).

In addition, the emergence of a distinct positive feature at 707.5 eV is attributed to the formation of Fe^3+^ polaron states, which are localized states arising from lattice distortion around the iron site. Polaron formation is typically accompanied by a local lattice expansion and electron self‐trapping, which can significantly influence carrier mobility and lifetime.^[^ ^37^ ^]^ Such behavior has been widely reported in related transition‐metal oxides.^[^ ^12^, ^13^, ^38^ ^]^ Therefore, the early‐time dynamics involve both electronic transitions and coupled electron–phonon interactions, which contribute to the observed spectral modifications.

#### 2.3.3 Intermediate Relaxation (1 ps)

At 1 ps, the system begins to relax toward its original configuration. The CFS increases to 1.75 eV (Figure 3e), suggesting partial restoration of iron–oxygen orbital overlap and local symmetry. Most high‐spin and intermediate‐spin Fe^2+^ states have decayed by this time, as evidenced by the diminished intensity ≈705.5 eV. The remaining spectral intensity is attributed predominantly to non‐excited Fe^3+^ centers in a nearly relaxed environment. The partial recovery of the crystal field suggests that some electron‐hole recombination and structural reordering have occurred, though the system has not yet fully returned to equilibrium.

#### 2.3.4 Long‐Term Relaxation (5 ps)

By 5 ps, the system exhibits a CFS of 1.8 eV (Figure 3f), nearly identical to the ground state value, indicating that the electronic and structural relaxation is almost complete. Most photoexcited carriers have recombined, and the lattice distortions introduced by excitation have largely subsided. The calculated spectra suggest that the dominant residual signal at 707.5 eV corresponds to polaronic Fe^3+^ states, which persist even at later times.

In Figure 3b, the observed behavior of Peak D (*t_2g_*‐related) and Peak E (*e_g_*‐related) can be explained by the distinct dynamics of the electronic states and their relaxation pathways.

#### 2.3.5 Initial Sub‐Picosecond Decay

Both the *t_2g_* and *e_g_* features exhibit a rapid intensity drop on the sub‐picosecond timescale, with decay constants of ≈800 fs for Peak D and 900 fs for Peak E. This initial decay phase is attributed to the ultrafast relaxation of photoexcited charge carriers, specifically electrons in iron 3*d* orbitals and holes in oxygen 2*p* orbitals. The fast decay is most likely driven by strong electron–phonon coupling, where the excess energy from electronic excitation is rapidly transferred to the lattice via phonon emission. Since both *t_2g_* and *e_g_* orbitals participate in bonding and share similar orbital symmetries with the oxygen ligands in the octahedral field, their initial coupling to phonons may be comparable, resulting in similar early‐time decay constants.

#### 2.3.6 Long‐Term Decay Dynamics

In contrast, the long‐time behavior diverges significantly; Peak D, related to the *t_2g_* state, decays with a time constant of 100 ps, while Peak E, related to the *e_g_* state, persists for a much longer duration, with a decay constant of 850 ps. This pronounced difference suggests distinct relaxation pathways and decoupled long‐term dynamics for the two orbital manifolds. The distinct behavior of Peaks D and E reflects the orbital‐dependent nature of the relaxation processes, demonstrating how different iron 3*d* states interact with lattice, spin, and orbital degrees of freedom on ultrafast and longer timescales.

After the initial fast relaxation, the remaining excited *t_2g_​* electrons may quickly relax back to the ground state by coupling to lattice vibrations (phonons) or through non‐radiative recombination processes. The significantly longer decay time for the *e_g_* peak suggests that excited *e_g_* electrons are less efficiently coupled to the lattice and may require a longer relaxation time. This could be due to weaker electron‐phonon coupling for the *e_g_* ​ states or the involvement of more complex relaxation processes. These could include trapping carriers in metastable states or slower recombination dynamics. Alternatively, the longer *e_g_* ​ lifetime could also be due to slower relaxation pathways associated with spin or orbital relaxation processes, as the *e_g_* ​ states might exhibit a different interaction with the spin or orbital degrees of freedom compared to *t_2g_* ​ states. These observations could stem from other effects, such as metastable state formation and spin‐orbital dynamics discussed in Section 5.3 (Supporting Information).^[^ ^39^, ^40^, ^41^, ^42^ ^]^

### 2.4 Shifts and Changes in the XAS Intensities

**Figure 4:**
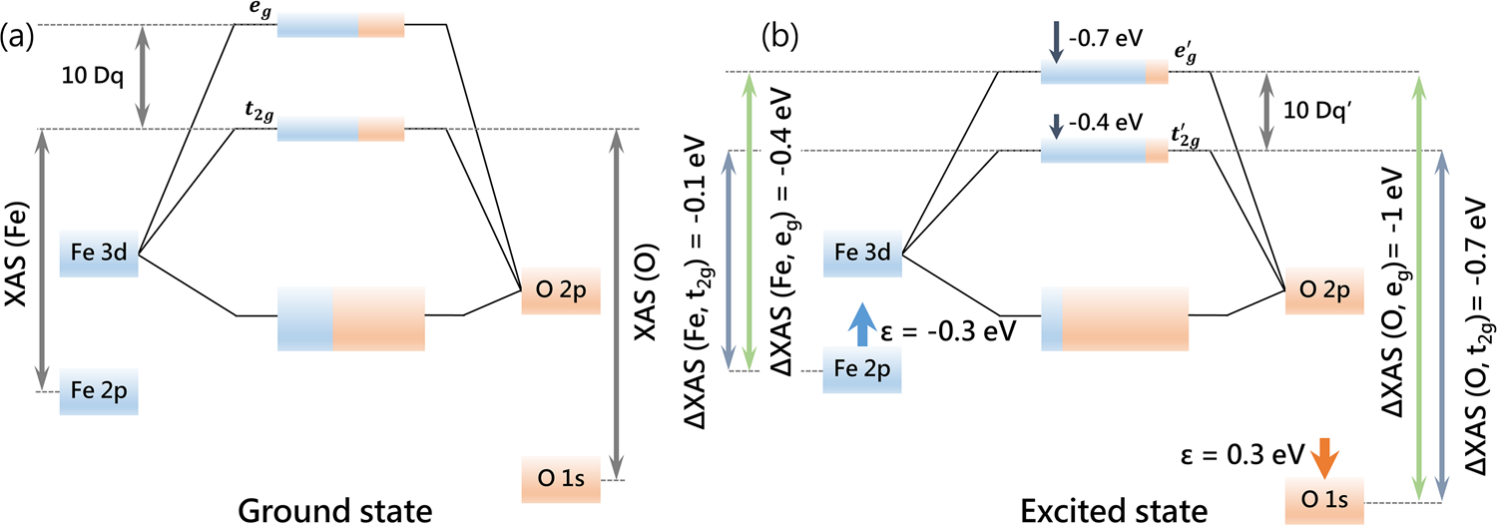
Energy diagrams for LaFeO_3_ a) ground state b) the energy shifts in the excited state, with −0.4 and −0.7 eV, for the *t_2g_* band and the *e_g_* band, respectively. The iron 2*p* XAS has reduced shifts (ε = −0.3 eV), while the oxygen 1*s* XAS has increased shifts (ε = 0.3 eV). As a result of the decreased overlap between the iron and oxygen orbitals, the valence band becomes increasingly dominated by the oxygen 2*p* character, while the iron 3*d* band is more strongly characterized by the iron 3*d* orbitals.

**Figure** **4** depicts the energy diagrams for the ground state and the excited state of LaFeO_3_ observed in Figures 1a and 3a. The final state of the iron 2*p* XAS includes an iron 2*p* core hole, which introduces an additional potential on the iron 3*d* states. This added potential localizes the iron 3*d* orbitals, reducing their overlap with the oxygen 2*p* states. As a result, the anti‐bonding states become less anti‐bonding and shift to lower energies (Figure 4b). This effect is more pronounced in the *e_g_* states, which are more anti‐bonding, thereby reducing the CFS between the *t_2g_* and *e_g_* levels. Although the iron 2*p* XAS is strongly influenced by the multiplet effects, the final‐state CFS is known to be ≈20% smaller than the value observed in optical measurements, bringing it down to ≈1.8 eV.^[^ ^43^ ^]^

The final state of the oxygen 1*s* XAS includes an oxygen 1*s* core hole that acts as an extra potential to the oxygen 2*p* states. This effect is similar to the influence of the iron 2*p* core hole on the iron 3*d* states, but it introduces an additional impact. In essence, the core hole potential also affects the empty 3*d* states, deforming the band structure and piling up the intensity at the bottom of each band (Figure 1e). Due to the sharp profile of the *t_2g_* band, its peak energy decreases only slightly after lifetime broadening. In contrast, the broader *e_g_* band experiences a more significant shift as the PDOS piles up at the bottom of the band. Together, these effects reduce the *t_2g_*–*e_g_* splitting by an additional 0.2 eV (with respect to the iron 2*p* core hole), bringing it down to 1.6 eV (Figure 2a).

In short, the experiment shows an *e_g_* band shift of −1.0 eV and a *t_2g_* band shift of −0.7 eV for the oxygen 1*s* XAS (Figure 2a) upon excitation by a 400 nm laser. In the case of iron 2*p* XAS, the *e_g_* band shifts −0.4 eV, and the *t_2g_* band shifts −0.1 eV (Figure 3a). These shifts can be understood as follows: An optical excitation transfers an electron from the oxygen 2*p* VB to the iron 3*d* band and reduces the overlap between the iron and oxygen orbitals, causing less bonding and less anti‐bonding mixed states. This implies that both the *t_2g_* and *e_g_* bands shift to lower energies than the ground state, as indicated in Figure 4a,b. Since the *e_g_* band is σ‐anti‐bonding, the effect is larger for the *e_g_* band, effectively reducing the crystal field splitting. Due to the reduced overlap between the iron and oxygen orbitals, the VB becomes more dominated by the oxygen 2*p* character, and the conduction band becomes more dominated by the iron 3*d* character. This implies that the charge density increases at the oxygen sites and decreases at the iron sites. A decreased charge density at the iron sites tends to shift the iron 2*p* XAS to higher energies. This is indicated with the ε value of −0.3 eV. This ε‐shift suggests that the iron 2*p* XAS peaks shift less than expected from the 3*d* levels by 0.3 eV. Conversely, the oxygen 1*s* XAS shifts to lower energies (ε = 0.3 eV), indicating that the shifts for the oxygen 1s XAS are increased by 0.3 eV. We conclude that the reduced overlap between the oxygen 2*p* and iron 3*d* orbitals can explain all the experimental observations: 1) the XAS shifts to lower energies, 2) the decrease in the crystal field splitting, and 3) the relatively larger shifts for the oxygen 1*s* XAS.

## 3 Conclusion

In this study, we employed the fs‐XAS technique at PAL‐XFEL to unravel light‐driven ultrafast electronic and structural changes in epitaxial LaFeO_3_ thin films. We demonstrated that the holes in LaFeO_3_ persist twice as long as those in Fe_2_O_3_, highlighting its potential as a promising candidate for oxygen evolution reaction. Moreover, the CFS in LaFeO_3_ is inextricably intertwined with the local charge density of the iron ions. Variations in the CFS can be related to changes in the charge density due to photoexcitation and relaxation effects. The intermediate CFS values of 1.5 eV at 0.2 ps and 1.75 eV at 1 ps obtained from the multiplet calculations describe these transient states captured by our fs‐XAS experiments. The photoexcitation initially induces high‐ and intermediate‐spin Fe^2+^ states through LMCT, followed by polaron formation at longer delay times. Upon optical excitation, an electron is moved from the oxygen 2*p* VB to the iron 3*d* band, effectively reducing the orbital overlap between iron and oxygen. This leads to a downward energy shift of both the *t_2g_* and *e_g_* bands relative to the ground state. These findings shed light on the dynamic coupling between local charge density changes and the CFS in LaFeO_3_. In particular, they provide valuable insights into the ultrafast processes governing charge transfer and polaron formation—key mechanisms for understanding the photoexcited behavior of related materials.

## 4 Experimental Section

### fs‐XAS Experiments

The fs‐XAS spectra were acquired at the Soft X‐ray Scattering and Spectroscopy (SSS) beamline at the Pohang Accelerator Laboratory X‐ray Free Electron Laser (PAL‐XFEL) in South Korea. All measurements were performed at room temperature and in the ultra‐high vacuum condition of 10^−8^ mbar. The resolution of the experiment was 120 fs, a similar value as in our previous experiments.^[^ ^4^, ^44^, ^45^ ^]^ The average number of acquisitions varied between 5 and 10 depending on the signal/noise ratio. The static XAS was measured each time to monitor for potential beam damage. The monochromatized X‐rays were normalized using an I_0_ monitor and focused onto the sample with Kirkpatrick–Baez mirrors (Figure 1a). The measurements were conducted in electron yield (EY) mode, detected by a micro‐channel plate (MCP) detector. The relative timing between the pump and probe was controlled via an optical delay line, and the spatial and temporal overlap (delay = 0) was aligned by observing fluorescence from a thin cerium‐doped yttrium aluminum garnet (Ce:YAG) crystal.

### Optical Pump

A Ti:sapphire laser with a wavelength of 400 nm (3.10 eV) and a pulse duration of 80 fs was focused on a spot with a full width at half maximum (FWHM) of 100 µm. The fluence of the pump laser was 13.7 mJ cm^−^^2^. Across the fluence range of 8–30 mJ cm^−^^2^, no significant changes were observed in the transient features or lifetime behavior. At fluences below 3.4 mJ cm^−^^2^, the signal induced in the transients was too weak to provide a sufficient signal‐to‐noise ratio. The fluences up to 30 mJ cm^−^^2^ did not cause any damage to the sample.

### Pulsed Laser Deposition (PLD)

The epitaxial LaFeO_3_ thin film was grown using pulsed laser deposition (PLD) with in situ RHEED at the *MESA+ Institute for Nanotechnology of the University of Twente*, in the Netherlands. The Nb:SrTiO_3_ (100) substrates purchased from CrysTec GmbH were used for the growth. The Nb:SrTiO_3_ substrate was TiO_2_ terminated, which has already been demonstrated to suppress the possible extraction of photocarriers generated in Nb:SrTiO_3_.^[^ ^46^, ^47^ ^]^ The O_2_ pressure during the growth was 0.01 mbar, the growth temperature was 700 °C, the fluence was 1.9 J cm^−2^, the target‐substrate distance was 5 cm, and the frequency was 2 Hz. The thickness of the sample was determined to be ≈30 nm (Figure S9).

### Reflection High‐Energy Electron Diffraction (RHEED) and Atomic Force Microscopy (AFM)

The growth was monitored in situ using reflection high‐energy electron diffraction (RHEED), as illustrated in Figure S8a–c (Supporting Information). The topography of the grown films was characterized by atomic force microscopy (AFM) using a Veeco Dimension Icon AFM in tapping mode in the air. The oscillating cantilever is a Tespa‐V2 cantilever (Bruker, Netherlands) with a silicon tip with a nominal radius of 20 nm. The AFM images were obtained using the Nanoscope software and treated using the Gwyddion software,^[^ ^48^ ^]^ shown in Figure S8d (Supporting Information).

### High‐Resolution X‐Ray Diffraction (HRXRD)

The HRXRD characterization was carried out by a Bruker D8 Discover diffractometer with a high‐brilliance microfocus Cu rotating anode generator (2.5 kW), hybrid Montel optics, a two bounds monochromator, a 1 mm diameter circular pinhole beam collimator, and an EIGER2 R 500K hybrid photon counting area detector. The detector was operated in 0D mode with a region of interest of 13 × 65 pixels (75 × 75 µm^2^ each) for the 2θ‐ω scan (Figure S9a). The reciprocal space map (RSM) was obtained with the detector in 1D mode while performing a rocking curve (Figure S9b).

### Density Functional Theory (DFT) Calculations

The DFT calculations were performed using the QuantumESPRESSO package.^[^ ^49^, ^50^, ^51^, ^52^ ^]^ The wavefunctions were expanded with plane waves up to an energy cut‐off of 80 Ry and a 6×4×6 Monkhorst‐Pack grid was used to sample the Brillouin Zone. PBE‐GGA functional was used to describe the exchange‐correlation potential, and correlations for Fe *d* and La *f* orbitals were corrected using Hubbard parameters as described in ref. [29]. X‐ray absorption spectra were calculated using the XSpectra code (Figure S2).^[^ ^52^, ^53^, ^54^ ^]^

### Multiplet Calculations

The multiplet calculations were carried out using CTM4XAS^[^ ^55^ ^]^ and Quanty.^[^ ^56^, ^57^, ^58^ ^]^

## Conflict of Interest

The authors declare no conflict of interest.

## Author Contributions

M.L., U.B., and F.M.F.d.G. conceived the experiments. S.H.P., A.K., and S.K. designed and carried out the fs‐XAS experiments. M.L. and S.H.P. analyzed the fs‐XAS data. F.J.M., H.J.F.A.B., M.L., and F.M.F.d.G. performed the multiplet calculations and interpreted the fs‐XAS data. A.B.M. conducted the DFT calculations. A.B.M., M.L., and F.M.F.d.G. interpreted the DFT calculations. M.L., with the assistance of E.v.d.M., Y.A.B., and I. C. G. v.d.B. prepared the thin film samples using PLD. Y.A.B. and E.M.K. performed the HRXRD and RSM measurements. Y.A.B. simulated and interpreted the HRXRD and RSM data. C.B. and G.K. supervised the sample preparation. M.L. and F.M.F.d.G. wrote the manuscript with contributions from all the co‐authors.

## Supporting information



Supporting Information

## Data Availability

The data that support the findings of this study are openly available in DataverseNL in ref. [59].
