# Supplementary material for: Ultrafast Light‐Driven Electronic and Structural Changes in LaFeO3 Perovskites Probed by Femtosecond X‐Ray Absorption Spectroscopy
Source: Adv Mater. 2025 May 15;37(29):2502932. doi: 10.1002/adma.202502932 (PMC12288815; doi:10.1002/adma.202502932)
Supplement: Supplementary file 1 — Supporting Information [file ADMA-37-2502932-s001.pdf]

ADVANCED MATERIALS

Supporting Information

for *Adv. Mater.*, DOI 10.1002/adma.202502932

Ultrafast Light-Driven Electronic and Structural Changes in $LaFeO_3$ Perovskites Probed by Femtosecond X-Ray Absorption Spectroscopy

Masoud Lazemi, Fabian J. Mohammad, Sang Han Park, Abhishek Katoch, Hans J.F.A. Blankesteijn, Andrés R. Botello-Méndez, Emma van der Minne, Yorick A. Birkhölzer, Iris C. G. van den Bosch, Ellen M. Kiens, Christoph Baeumer, Gertjan Koster, Soonnam Kwon, Uwe Bergmann and Frank M. F. de Groot**

Supplementary information

Ultrafast light-driven electronic and structural changes in $LaFeO_3$ perovskites probed by femtosecond X-ray absorption spectroscopy

Masoud Lazemi[1,2,*], Fabian J. Mohammad[1], Sang Han Park[3], Abhishek Katoch[4], Hans J.F.A. Blankesteijn[1], Andrés R. Botello-Méndez[1], Emma van der Minne[2], Yorick A. Birkhölzer[2], Iris C. G. van den Bosch[2], Ellen M. Kiens[2], Christoph Baeumer[2], Gertjan Koster[2], Soonnam Kwon[3], Uwe Bergmann[5], and Frank M. F. de Groot[1*]

[1]Materials Chemistry and Catalysis, Debye Institute for Nanomaterials Science, Utrecht University, Universiteitsweg 99, 3584 CG Utrecht, The Netherlands

[2]MESA+ Institute for Nanotechnology, University of Twente, P. O. Box 217, Enschede, 7500 AE, The Netherlands

[3]Pohang Accelerator Laboratory, Pohang, Gyeongbuk 37673, South Korea

[4]Department of Chemistry, Yonsei University, Seoul 03722, South Korea

[5]Department of Physics, University of Wisconsin–Madison, Madison, WI, USA

**Corresponding authors: m.lazemi@uu.nl and F.M.F.deGroot@uu.nl*

1. Oxygen 1*s* XAS

1.1. Qualitative analysis

Fig. S1 shows the ground state (GS) oxygen 1*s* XAS spectrum reported in Fig. 2a fitted with Voigt and sigmoid functions. The parameters for the fitting are reported in Table S1.

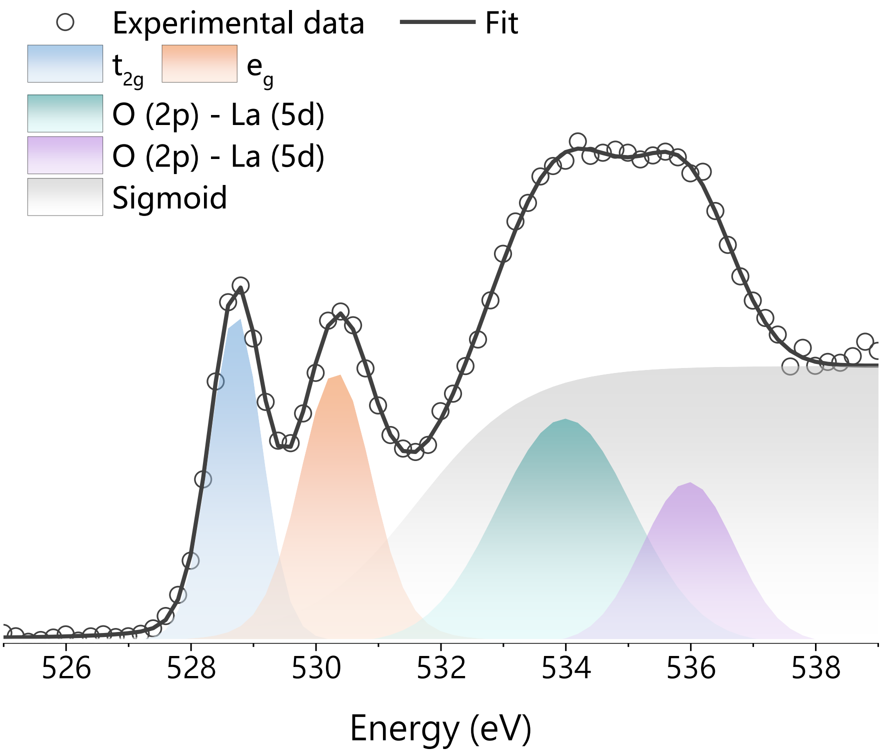

Fig. S1. (a) GS oxygen 1*s* XAS of $LaFeO_3$ fitted using Voigt and sigmoid functions. The vertical axis shows the intensity in arbitrary units. The sigmoid function is centered at 531.62 eV, with a slope of 0.87.

Table S1. Fitting parameters in Fig. S1

Peak	Energy (eV)	FWHM (eV)	Area
t_{2g}	528.72	0.98	26.27
e_g	530.31	1.33	27.23
O (2*p*) – La (5*d*)	533.97	2.58	49.23
O (2*p*) – La (5*d*)	535.97	1.74	22.83

1.2. DFT calculation of oxygen 1*s* XAS

Fig. S2 illustrates the oxygen 1*s* XAS simulation with different models using the XSPECTRA[1] code of QuantumESPRESSO package[2,3]. Fermi's golden rule for x-ray absorption cross-section is defined in Eq. S1.[4]

$$\sigma(\omega) = 4\pi^2\alpha_0\hbar\omega \sum_{f,\vec{k},\sigma} \left|\left\langle\psi^{\sigma}_{f,\vec{k}}\middle|\vec{e}\cdot\vec{r}\middle|\psi^{\sigma}_{f,\vec{i}}\right\rangle\right|^2 \delta(E^{\sigma}_{f,\vec{k}} - E^{\sigma}_{i,\vec{k}} - \hbar\omega) \qquad \text{(Eq. S1)}$$

In this equation, α_0 is the fine-structure constant, $\hbar\omega$ is the photon energy, $\vec{\boldsymbol{k}}$ represents the points in the Brillouin zone, σ is the spin state, $\psi^{\sigma}_{f,\vec{k}}$ and $\psi^{\sigma}_{f,\vec{i}}$ are the wavefunctions of the initial *i* and final *f* states, with corresponding energies of $E^{\sigma}_{i,\vec{k}}$ and $E^{\sigma}_{f,\vec{k}}$. The $\vec{\boldsymbol{e}}\cdot\vec{\boldsymbol{r}}$ is the transition operator in the dipole approximation where $\vec{\boldsymbol{e}}$ and $\vec{\boldsymbol{r}}$ are the polarization vector of the photon beam and the electron position vector. This code is implemented by evaluating in reciprocal space, based on results from DFT (or DFT + U, or DFT + U + V) calculations, utilizing the Lanczos recursive algorithm.

As shown in Fig. 2e, below the Fermi level, the projected density of states (PDOS) exhibits a more complex nature. In the absence of interactions between iron 3*d* electrons and electrons from the surrounding environment, the occupied iron 3*d* states would shift to lower energy by approximately U = 5 eV, while preserving the CFS. However, interactions with oxygen result in a mixed character of the iron 3*d* and oxygen 2*p* states in the valence band (VB). Fig. S2a shows that the DFT + U without a core hole captures the intensity of the e_g peak very well. It performs well for the 533 – 538 eV region, arising from oxygen (2*p*) – lanthanum (5*d*) anti-bonding states. Adding the core hole at different oxygen sites led to losing much of the e_g intensity but yielded a slight improvement in the 533 – 538 eV region for hole@O1, but not for hole@O2 (Fig. S2b). Table S2 reports the Hubbard parameters used in the calculations. The values were extracted from reference [4]. It should be noted that the precise simulation of oxygen 1*s* XAS of $LaFeO_3$ remains challenging, demanding further investigations.

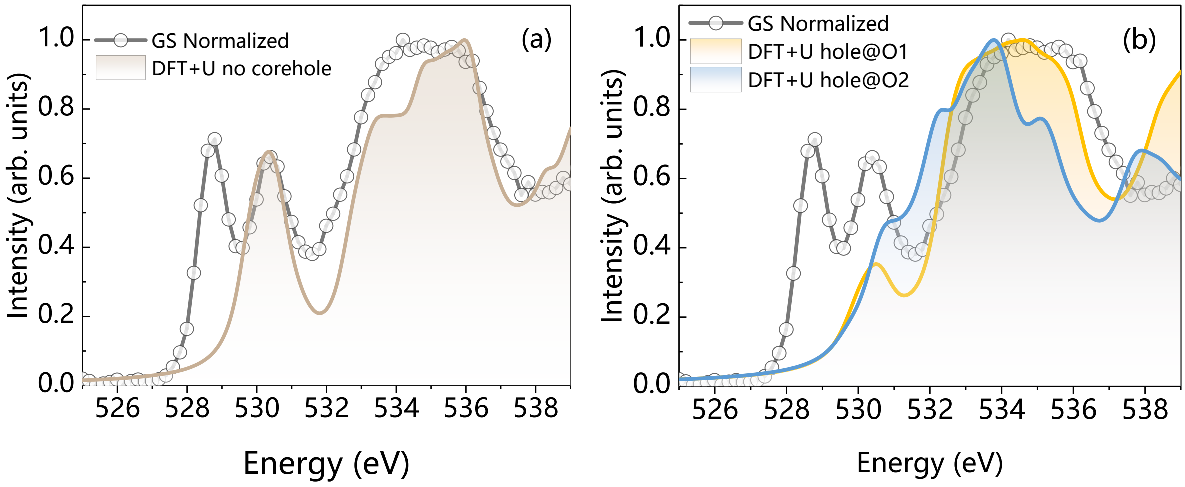

Fig. S2 Simulated oxygen 1*s* XAS (a) DFT+U no corehole (b) DFT+U hole@O1 and @ O2.

Table S2. Self-consistent Hubbard parameters for $LaFeO_3$ in eV

$U_{Fe(3d)}$	$U_{La(4f)}$	$V_{Fe(3d)-O(2p)}$
5.54	3.22	0.77

2. Excited state spectra

The excited spectrum, ES(λ, t), is believed to result from a combination of a rigid shift in the ground state spectrum, GS(λ, 0), and a change in crystal field splitting (10Dq). The rigid shift compared to the GS is attributed to a shift in the chemical potential (CPS, Δμ) and a core level shift (CES, ε) caused by excited electrons.[5,6] We have examined the photo-excitation process in detail to dissect these two effects. If the light absorption by the sample is less than 1 (i.e., α), the observed spectrum, OS(λ, t), after laser exposure should be a combination of the unaffected GS spectrum and the ES spectrum, as follows:

$$OS(\lambda, \mathrm{t}) = (1-\alpha)\, GS(\lambda, 0) + \alpha\, ES(\lambda, \mathrm{t}) = GS(\lambda, 0) + \alpha\, (ES(\lambda, \mathrm{t}) - GS(\lambda, 0)) \qquad \text{(Eq. S2)}$$

Since the transient spectrum, TR(λ, t), is defined as $OS(\lambda, \mathrm{t}) - GS(\lambda, 0)$, we obtain:

$$OS(\lambda, \mathrm{t}) - GS(\lambda, 0) = TR(\lambda, \mathrm{t}) = \alpha\, (ES(\lambda, \mathrm{t}) - GS(\lambda, 0)) \qquad \text{(Eq. S3)}$$

Therefore, we can derive the excited spectrum, ES(λ, t), from the two measured quantities, the ground state spectrum GS(λ, 0) and the transient spectrum TR(λ, t), as follows:

$$ES(\lambda, \mathrm{t}) = \frac{1}{\alpha} TR(\lambda, \mathrm{t}) + GS(\lambda, 0) \qquad \text{(Eq. S4)}$$

The absorption ratio, α, can be determined based on the assumption that at every energy λ the excited spectrum, ES, is greater than zero.

$$ES(\lambda, \mathrm{t}) = \frac{1}{\alpha} TR(\lambda, \mathrm{t}) + GS(\lambda, 0) > 0 \qquad \text{(Eq. S5)}$$

Fig. S3(a-d) show the oxygen 1*s* XAS spectra, illustrating GS and ES spectra at 0.2 ps, 1 ps, and 5 ps, with different values of α = 0.15, 0.2, 0.25, and 0.3. The α = 0.1 spectra are plotted in the manuscript (Fig. 2a).

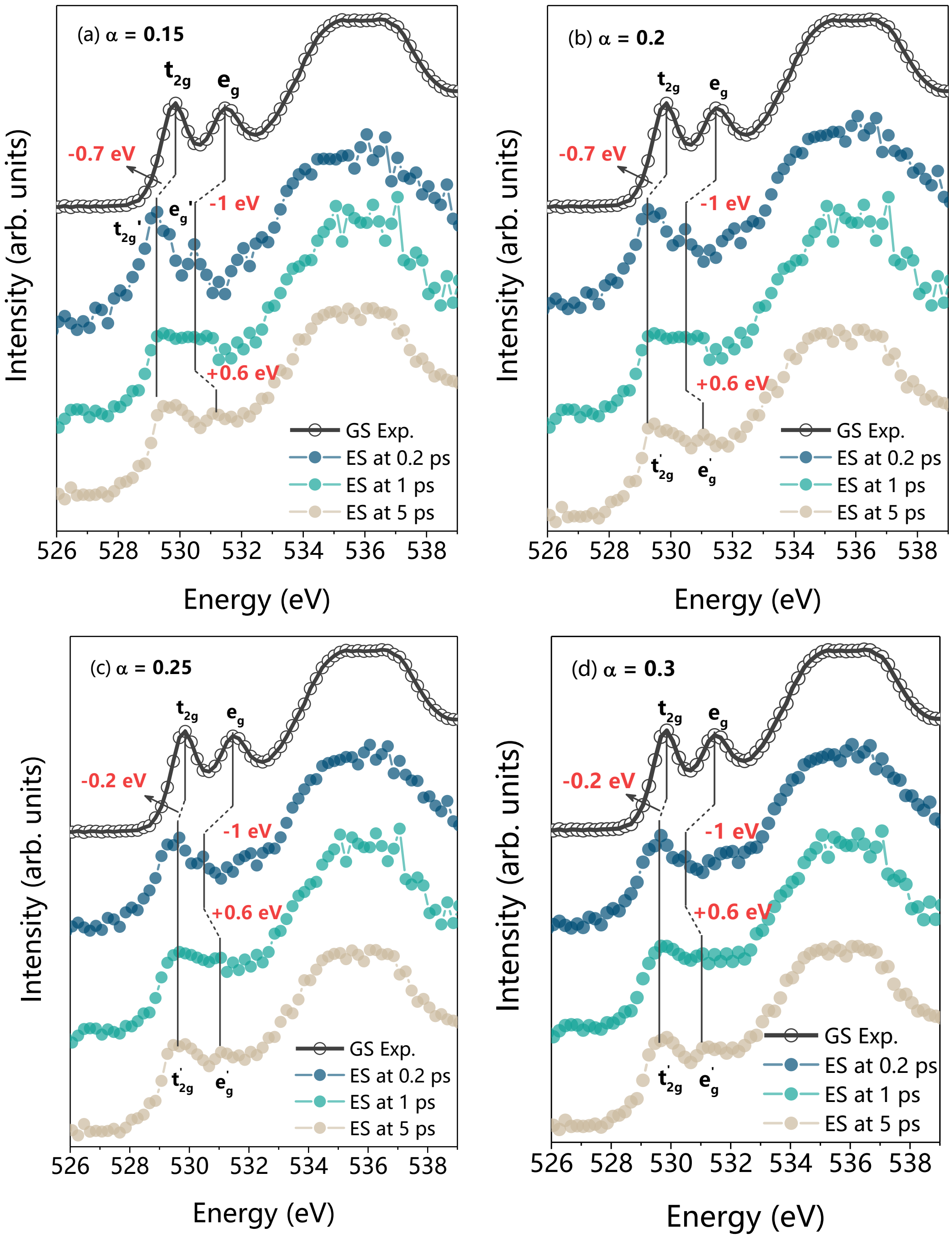

Fig. S3 Comparative analysis of oxygen 1*s* fs-XAS of $LaFeO_3$, illustrating GS and ES spectra at 0.2 ps, 1 ps, and 5 ps. The ES spectra were derived using Eq. S5. (a) α = 0.15, (b) α = 0.2, (c) α = 0.25, and (d) α = 0.3.

3. Fitting kinetic traces

The following formula was used to fit the kinetic traces[5–7]:

$$I(\Delta t) = \frac{1}{2}\left(1 - erf\left(\frac{-(\Delta t - t_{cref})}{\sqrt{2}\frac{\tau_{ref}}{2.355}}\right)\right)\left(I_1 exp\left(-\frac{\Delta t - t_{c1}}{\tau_1}\right) + I_2 exp\left(-\frac{\Delta t - t_{c2}}{\tau_2}\right) + I_3 exp\left(-\frac{\Delta t - t_{c3}}{\tau_3}\right)\right), \quad \text{(Eq. S6)}$$

where I_1, I_2, I_3, τ_1, τ_2, and τ_3 are the amplitude and the relaxation time constants. τ_{ref} is the time constant for the error function (erf).

4. fs-XAS at La 3*d* edge

The La M_4 edge XAS (Fig. S4) corresponds to the transitions from the 3d core levels to the 4f.

- **Electronic configuration and sensitivity:** La in the compound $LaFeO_3$ typically has a +3 oxidation state, resulting in a $4f^0$ electronic configuration.
- **Localized excitation effects:** The 400 nm laser excites electrons across the band gap (~2 eV), primarily involving iron 3*d* and oxygen 2*p* states. These states are not strongly hybridized with the La 4*f* states.

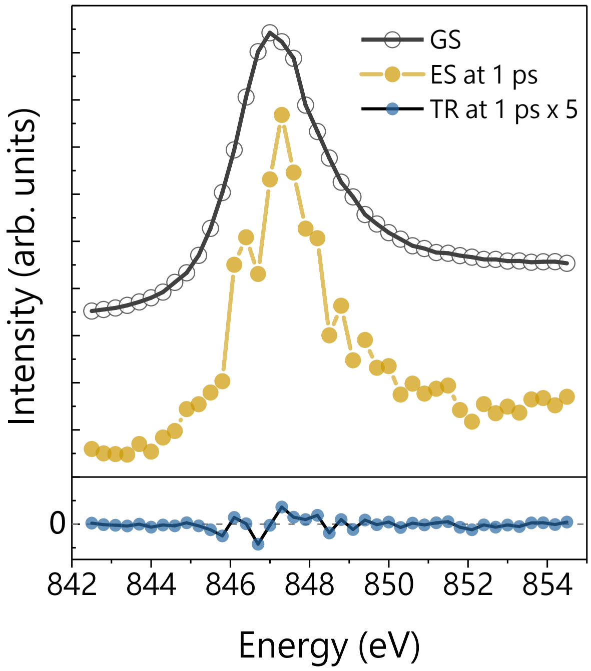

Fig. S4 La M_4 edge GS alongside ES (α = 0.1) and TR spectra at 1 ps.

5. Analyses of iron 2*p* XAS

5.1. Simulated ground-state iron 2*p* XAS

Fig. S5 compares the experimental and simulated GS spectra using CTM4XAS[8]. It is noteworthy that the simulated GS spectrum perfectly correlates with the experimental data. The 10Dq was set to 1.8 eV, and we used the O_h symmetry. The Slater integrals (F_{dd}, F_{pd}, G_{pd}) were reduced by 80% of the Hartree–Fock (HF) values. The core and valence spin-orbit coupling were taken into account in the calculations. We used a Gaussian broadening of 0.2 eV. The Lorentzian broadening E < 707.8 eV was set to 0.28 eV, while it was increased to 0.58 eV for E > 707.8 eV to account for the lifetime of the states. The parameters used in the calculations are reported in Table S3.[9] The Fe^{3+} has a $^6A_2$ symmetry, resulting in a negligible effect of spin-orbit coupling on the simulations.

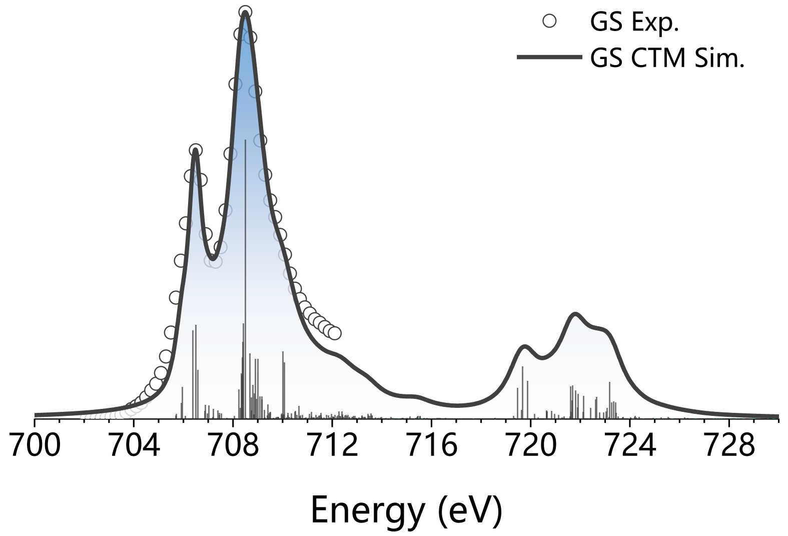

Fig. S5 Simulated GS iron 2p XAS compared with experimental data. The sticks are representative of atomic multipltes calculated by CTM4XAS. The y-axis is the intensity in arbitrary units.

Table S3 The parameters used in the calculations, Slater integrals, and spin-orbit couplings

	F^2_{dd}	F^4_{dd}	ζ_{2p}	F^2_{pd}	G^1_{pd}	G^3_{pd}
$2p^6 3d^5$	12.043	7.535				
$2p^5 3d^6$	12.818	8.023	8.199	7.446	5.566	3.166
$2p^6 3d^6$	10.965	6.815				
$2p^5 3d^7$	11.778	7.327	8.200	6.792	5.000	2.843
$2p^6 3d^4$	13.030	8.198				
$2p^5 3d^5$	13.776	8.661	8.199	8.103	6.153	3.503

5.2. Calculation of iron $2p_{3/2}$ excited state and transient spectra

Upon photoexcitation by laser, a valence electron is promoted to the conduction band, corresponding to a $|3d^6\underline{L}\rangle$ state. For simplicity, we approximate the ground state and laser-excited state as $|3d^5\rangle$ and $|3d^6\underline{L}\rangle$, while noting that the optically excited state has opposite parity. The state $|3d^6\underline{L}\rangle$ represents a configuration where one electron is transferred from oxygen 2*p* to iron 3d. This charge-transfer state has an associated energy (Δ) that differs from the band gap due to strong electron-correlation effects. The effect of the ligand hole ($\underline{L}$) in the ground state was not considered in the calculations. For the ES spectra (Fig. 3b and S6), the spin-orbit coupling was switched off since $|3d^6\rangle$ has 6 sub-states, which are very close in energy. Therefore, the laser can excite the electron to all these sub-states. Moreover, the spin-orbit coupling is quenched by band structure and dispersion. The polaron state was simulated assuming D_s and D_t distortions of 0.2 eV in the D_{4h} symmetry. The detailed description of the parameters can be found in ref [8,10].

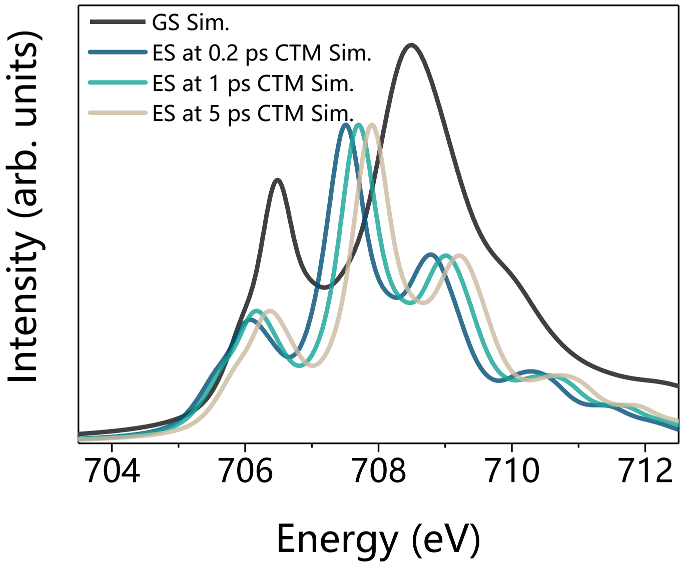

Fig. S6 Calculated GS and ES spectra. The corresponding TR spectra are shown in Fig. 3b.

5.3. Kinetic traces at iron 2$p_{3/2}$ edge

As mentioned in the manuscript (Section 2), the different behaviors for Peak D and E in Fig. 3b could be attributed to the following aspects:

- **Metastable state (polaron) formation:** The longer lifetime of the e_g state could also indicate the formation of metastable states where excited electrons become temporarily trapped in localized states. These states could slowly decay via non-radiative processes, leading to a longer relaxation time of 800 ps.
- **Spin-orbital dynamics:** In transition metal oxides like $LaFeO_3$, the relaxation of excited states can also be influenced by spin-orbit coupling or spin-lattice relaxation. If the e_g state involves a slower spin relaxation process than the t_{2g} state, this could also account for the longer lifetime observed for e_g.

5.4. Iron 2$p_{3/2}$ ES spectra with different α values

Fig. S7(a-d) compare the iron $2p_{3/2}$ XAS spectra, illustrating GS and ES spectra at 0.2 ps, 1 ps, and 5 ps, with different values of α = 0.15, 0.2, 0.25, and 0.3. The α = 0.1 spectra are plotted in the manuscript (Fig. 3a). (α was defined in SI section 2)

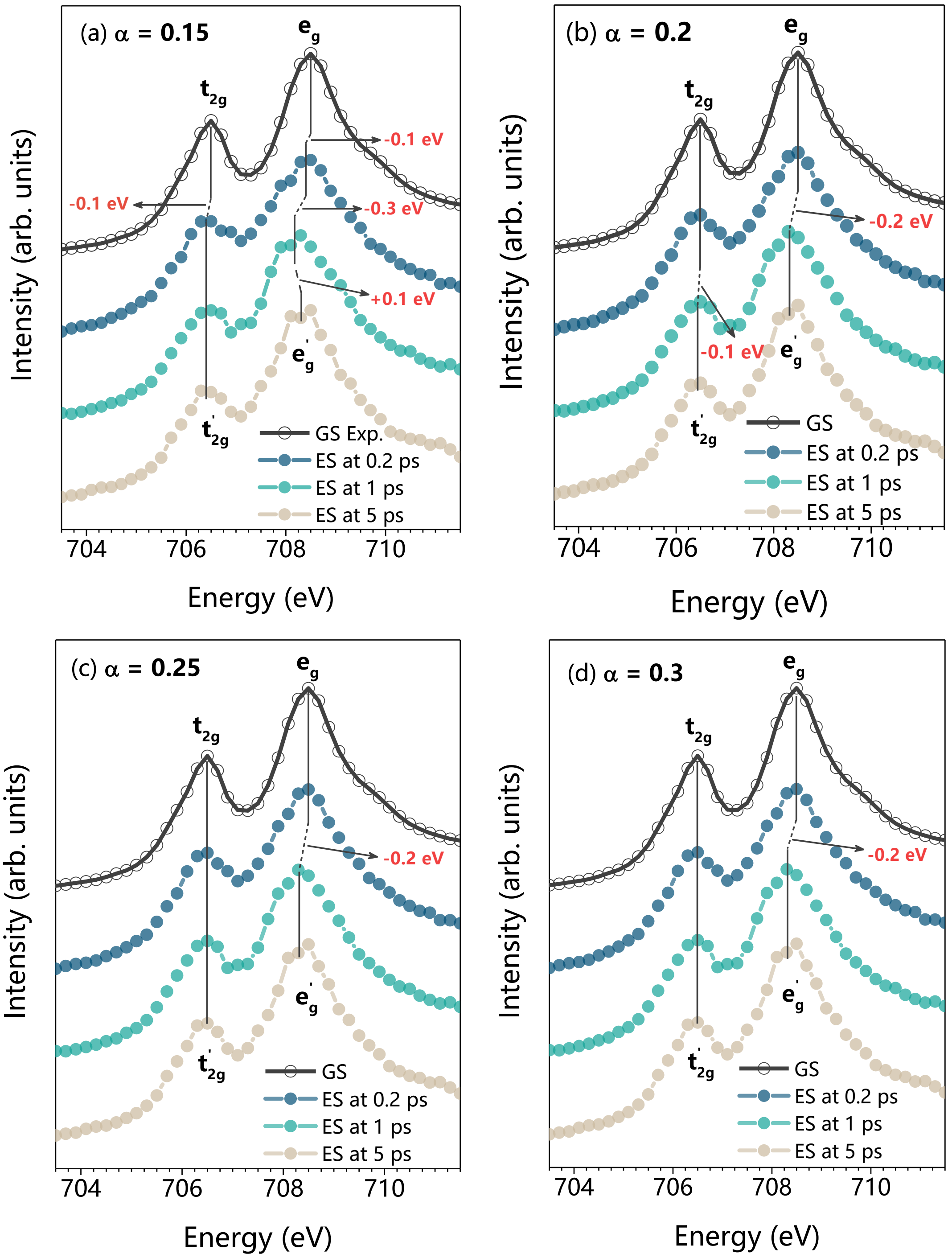

Fig. S7 Comparative analysis of iron $2p_{3/2}$ fs-XAS of $LaFeO_3$, illustrating GS and ES spectra at 0.2 ps, 1 ps, and 5 ps. The ES spectra were derived using Eq. S5. (a) α = 0.15, (b) α = 0.2, (c) α = 0.25, and (d) α = 0.3.

6. Reflection high-energy electron diffraction (RHEED) and atomic force microscopy (AFM)

Fig. S8a illustrates the reflection high energy electron diffraction (RHEED) pattern of the (100) Nb:$SrTiO_3$ substrate prior to starting $LaFeO_3$ deposition. The pattern exhibits a high-intensity specular spot, Bragg spots, and Kikuchi lines. After growth (Fig. S8b), the pattern reveals narrow streaks, indicating high crystallinity and relatively smooth, flat surfaces. The intensity of the specular spot was monitored during the growth (Fig. S8c), with the arrow indicating the completion of the deposition. Notably, the layer-by-layer growth of the thin film was confirmed by the consistent RHEED oscillations. The oscillations maintained their amplitude up to around 30 u.c., after which they gradually diminished until disappearing. This sustained oscillation before fading enables precise in-situ determination of the growth rate, allowing the deposition to be stopped at any desired thickness with high accuracy. The vertical spike (around 340 s) occurs when one increases the filament current to get more signals to compensate for increasing surface roughness and drift. The AFM image (Fig. S8d) clearly displays the evenly-spaced surface steps after deposition, consistent with the predominant layer-by-layer growth mode and minimal island formation.

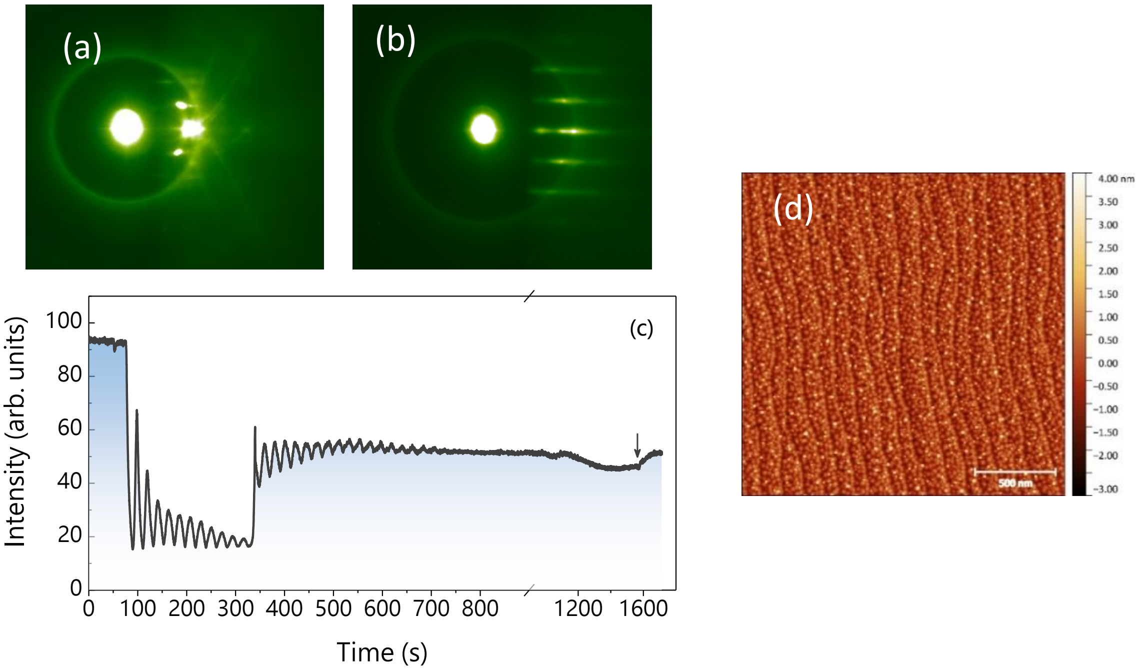

Fig. S8 The RHEED images (a) before growth (b) after growth (c) RHEED intensity oscillations during the growth. (d) AFM image after growth.

7. X-ray diffraction (XRD) and reciprocal space mapping

In Fig. S9a, the simulated X-ray diffraction (XRD) data resulted in 28.8 nm thickness of $LaFeO_3$. Moreover, we performed X-ray reciprocal space mapping of an asymmetric reflection (Fig. S9b). We chose the vicinity of the 103 peak of the Nb:$SrTiO_3$ substrate and collected the data in grazing-exit configuration. All the intensity from the film sits at exactly the same in-plane momentum q_x as the substrate, i.e., horizontal coordinate. Hence, the $LaFeO_3$ film is commensurately strained to the substrate. The elongated shape along the q_z (vertical) axis is a finite-size effect. A relaxed or highly defective film would show intensity at q_x values other than the same as the substrate, e.g., a diagonally shifted peak, or one with a heavy tail towards the left or right. It is noteworthy that the high-quality growth of $LaFeO_3$ thin film was confirmed by RHEED, AFM, and HRXRD characterizations (Fig. S8 and S9).

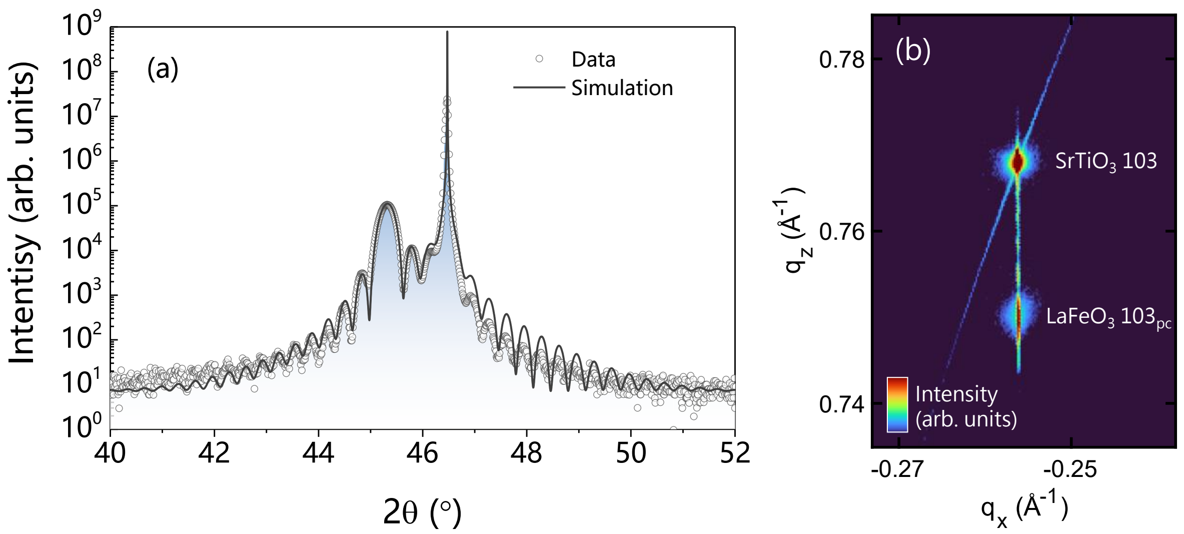

Fig. S9 (a) 2θ – ω scan with superimposed fit (b) reciprocal space mapping in the vicinity of the 103 peak of the Nb:$SrTiO_3$ substrate.

References:

1. Taillefumier, M., Cabaret, D., Flank, A.-M. & Mauri, F. X-ray absorption near-edge structure calculations with the pseudopotentials: Application to the *K* edge in diamond and α -quartz. *Phys. Rev. B* **66**, 195107 (2002).
2. Giannozzi, P. *et al.* QUANTUM ESPRESSO: a modular and open-source software project for quantum simulations of materials. *J. Phys.: Condens. Matter* **21**, 395502 (2009).
3. Giannozzi, P. *et al.* Advanced capabilities for materials modelling with Quantum ESPRESSO. *J. Phys.: Condens. Matter* **29**, 465901 (2017).
4. Timrov, I. *et al.* Electronic structure of pristine and Ni-substituted $LaFeO_3$ from near edge x-ray absorption fine structure experiments and first-principles simulations. *Phys. Rev. Research* **2**, 033265 (2020).
5. Park, S. H. *et al.* Direct and real-time observation of hole transport dynamics in anatase TiO_2 using X-ray free-electron laser. *Nat Commun* **13**, 2531 (2022).
6. Katoch, A. *et al.* Nano-Size Effects on Decay Dynamics of Photo-Excited Polarons in CeO_2. *Advanced Optical Materials* 2401386 (2024) doi:10.1002/adom.202401386.
7. Miao, T. J. & Tang, J. Characterization of charge carrier behavior in photocatalysis using transient absorption spectroscopy. *The Journal of Chemical Physics* **152**, 194201 (2020).
8. Stavitski, E. & de Groot, F. M. F. The CTM4XAS program for EELS and XAS spectral shape analysis of transition metal L edges. *Micron* **41**, 687–694 (2010).
9. Haverkort, M. W. Spin and orbital degrees of freedom in transition metal oxides and oxide thin films studied by soft x-ray absorption spectroscopy. Preprint at https://doi.org/10.48550/ARXIV.COND-MAT/0505214 (2005).
10. Groot, F. de. Multiplet effects in X-ray spectroscopy. *Coordination Chemistry Reviews* **249**, 31–63 (2005).
